# Pulmonary Artery Denervation Reduces Pulmonary Artery Pressure and Induces Histological Changes in an Acute Porcine Model of Pulmonary Hypertension

**DOI:** 10.1161/CIRCINTERVENTIONS.115.002569

**Published:** 2015-11-17

**Authors:** Alexander M.K. Rothman, Nadine D. Arnold, William Chang, Oliver Watson, Andrew J. Swift, Robin Condliffe, Charlie A. Elliot, David G. Kiely, S. Kim Suvarna, Julian Gunn, Allan Lawrie

**Affiliations:** From the Department of Cardiovascular Science (A.M.K.R., N.D.A., O.W., S.K.S., J.G., A.L.), Academic Unit of Radiology (A.J.S.), and INSIGNEO, Institute for Insilico Medicine (A.M.K.R., A.J.S., J.G.), University of Sheffield, Sheffield, United Kingdom; Medtronic Inc, Santa Rosa, CA (W.C.); and Sheffield Pulmonary Vascular Disease Unit, Royal Hallamshire Hospital, Sheffield, United Kingdom (R.C., C.A.E., D.G.K.).

**Keywords:** ablation techniques, hypertension, pulmonary, nervous system, pulmonary artery, pulmonary circulation

## Abstract

Pulmonary arterial hypertension is a devastating disease with high morbidity and mortality and limited treatment options. Recent studies have shown that pulmonary artery denervation improves pulmonary hemodynamics in an experimental model and in an early clinical trial. We aimed to evaluate the nerve distribution around the pulmonary artery, to determine the effect of radiofrequency pulmonary artery denervation on acute pulmonary hypertension induced by vasoconstriction, and to demonstrate denervation of the pulmonary artery at a histological level.

**Methods and Results—:**

Histological evaluation identified a circumferential distribution of nerves around the proximal pulmonary arteries. Nerves were smaller in diameter, greater in number, and located in closer proximity to the luminal aspect of the pulmonary arterial wall beyond the pulmonary artery bifurcation. To determine the effect of pulmonary arterial denervation acute pulmonary hypertension was induced in 8 pigs by intravenous infusion of thromboxane A_2_ analogue. Animals were assigned to either pulmonary artery denervation, using a prototype radiofrequency catheter and generator, or a sham procedure. Pulmonary artery denervation resulted in reduced mean pulmonary artery pressure and pulmonary vascular resistance and increased cardiac output. Ablation lesions on the luminal surface of the pulmonary artery were accompanied by histological and biochemical alteration in adventitial nerves and correlated with improved hemodynamic parameters.

**Conclusions—:**

Pulmonary artery denervation offers the possibility of a new treatment option for patients with pulmonary arterial hypertension. Further work is required to determine the long-term efficacy and safety.

## Abstract 2

Supplemental Digital Content is available in the text.

**WHAT IS KNOWN**

- Sympathetic tone is increased in patients with pulmonary arterial hypertension.
- Increased sympathetic tone has been associated with elevated pulmonary artery pressures.

**WHAT THE STUDY ADDS**

- Documents the distribution and depth of nerves surrounding the pulmonary artery in an animal model.
- Demonstrates the acute effect of radiofrequency energy delivery to the pulmonary artery wall.
- Demonstrates the efficacy of pulmonary artery denervation in an acute model of pulmonary hypertension.

Pulmonary arterial hypertension (PAH) comprises a range of diseases defined by a resting mean pulmonary artery pressure (PAP) of ≥25 mm Hg.^1^ Disease pathology is characterized by vasoconstriction and pulmonary vascular remodeling. Treatment options are limited to pharmacological vasodilatation via the prostacyclin, endothelin or nitric oxide pathways, or lung transplantation.^2^ Although there have been significant improvements in outcome over the past 2 decades, PAH remains a life shortening condition and survival at 3 years is 68%.^1^ There is a clear and pressing need for novel treatment strategies.

**See Editor’s Perspective by Leopold**

Autonomic regulation of pulmonary vasculature tone is well recognized, but its role in PAH is less clearly defined. Increased plasma norepinephrine,^3,4^ muscle sympathetic nerve activity,^5,6^ and vessel sympathetic nerve endings^7^ have been demonstrated in patients with idiopathic PAH, identifying the neurohormonal axis as a potential therapeutic target.

The sympathetic hyperactivity observed in patients with PAH is partially chemoreflex mediated,^5^ but the contribution of other physiological mechanisms cannot be excluded.^8^ Baroreceptors at the pulmonary artery bifurcation and along the large pulmonary arteries activate afferent fibers carried in the adventitia, which together with effector fibers in the muscle layer form a pulmo-pulmonary baroreceptor reflex.^9^ Surgical disruption of these fibers has been shown to prevent rises in PAP induced by balloon occlusion^8,10^; however, the relative contribution of basal sympathetic tone and baroreceptor stimulation to effector fiber activity is unclear. The development of percutaneous methods of arterial denervation now permits investigation of pulmonary artery denervation (PDN) as a treatment for PAH. The benefit of PDN has been shown in preclinical^11^ and early clinical studies.^12^ The distribution of nerves around the pulmonary arteries, the mechanism of action, and the effect of radiofrequency ablation on the pulmonary artery wall are unknown. The immediate changes in PAP observed in preclinical^11^ and clinical studies^12^ of PDN cannot be explained by altered remodeling of the distal pulmonary arteries which therefore suggests an effect on vascular constriction. We therefore aimed to examine nerve distribution around the pulmonary arteries, demonstrate the effect of PDN in an acute vasoconstrictive model of pulmonary hypertension (PH), and to show histological evidence of radiofrequency ablation on the nerves within the pulmonary artery adventitia.

## Methods

### Anatomic Distribution of Nerves Surrounding the Pulmonary Artery

To determine the anatomic relationship of nerve tissue to the pulmonary artery, histological samples were prepared from Yorkshire white swine. Animals were sedated by intramuscular injection of azaperone (6–8 mg/kg) and euthanized by intravenous injection of phenobarbital (40 mg/kg) in accordance with The Animals (Scientific Procedures) Act 1986 under UK Home Office Project License 40/3722. The chest was opened via a midline thoracotomy and the pleura and pericardium dissected to expose the heart and great vessels. The inferior vena cava, superior vena cava, and descending aorta were cross-clamped, and the heart and lungs were mobilized and excised en bloc (Figure IA and IB in the Data Supplement).

For anatomic studies of nerve distribution, the pulmonary arteries were excised with local structures in place (Figure IA–IG in the Data Supplement). Tissue was washed in PBS and fixed overnight in 10% formalin. Axial tissue sections were cut post fixation, dehydrated in graded alcohols, and paraffin embedded. Five micrometer sections were mounted and stained with hematoxylin and eosin and S100 protein (Z0311, Dako). Blocks were trimmed to obtain representative axial sections of the main pulmonary artery and the left and right pulmonary arteries beyond the bifurcation (Figure IC–IG in the Data Supplement). Anatomic orientation was determined from local structures. Images were acquired on a scanning microscope (10× objective, Axiocam 506 Color, Zeiss, Germany) and analysis performed in Zen 2 Blue Edition (Zeiss, Germany). Each artery was divided into 4 equal anatomic quadrants (anterior, posterior, medial and lateral, or left and right as appropriate), and S100 and hematoxylin and eosin–stained sections used to identify nerves (Figure IE–IG in the Data Supplement). Diameter, XY coordinate (X_n_, Y_n_), and distance from luminal aspect of the pulmonary artery wall (d) were measured for each nerve. A single reference coordinate (X_r_, Y_r_) was plotted for each quadrant. The angle of the nerve from the reference point was calculated as θ=tan^−1^((Y_n_–Y_r_)/(X_n_–X_r_)). The nerve distribution of each quadrant was plotted as (θ, d) using the polar scatter plot function of the Plotly package (https://github.com/ropensci/plotly; http://plot.ly) in the open source programming language R (http://www.R-project.org/). Nerve distribution plots of idealized vessels were constructed from quadrant distribution plots in Photoshop CS6 (Adobe) at the level of the main pulmonary artery and the left and right pulmonary artery.

### Interventional Experimental Study Design

Eight Yorkshire white pigs (21–27.5 kg) were assigned to PDN or sham procedure. Under anesthesia (intramuscular azaperone, 40 mg/mL at 6 mg/kg; intravenous propofol, 10 mg/mL at 3 mg/kg; and isoflurane, 2% to 3% in 100% O_2_ via endotracheal tube), left and right heart catheterizations were performed with radiographic guidance (BV Pulsera, Philips). Following baseline hemodynamic measurements, stable thromboxane A_2_ agonist^13^ (TxA_2_, D0400, Sigma-Aldrich) was infused via a 6F sheath in the right internal jugular vein. The dose of infused TxA_2_ was increased at 5-minute intervals in accordance with defined protocol (10 μg/mL at 17, 22, 27, 32, and 37 μg/kg per hour) determined from preliminary experiments (data not shown). Each animal received the same escalating dose of TxA_2_ at the same time interval. Once maximum PAP was attained, TxA_2_ was discontinued. When PAP returned to baseline, PDN or a sham procedure was performed. To assess the efficacy of PDN, the infusion of TxA_2_ was repeated post procedure using the same TxA_2_ challenge protocol.

### Hemodynamic Measurements

Pulmonary hemodynamics were monitored via Swan Ganz catheter (Baxter Healthcare) and systemic arterial pressures via a 6F multipurpose catheter (Medtronic Inc). Cardiac output (CO) was measured by thermodilution and pulmonary vascular resistance (PVR) calculated as PVR=(mean PAP−LVEDP)/CO.

### PDN and Sham Procedure

PDN was performed using a modified prototype radiofrequency catheter and G2 generator (Medtronic Inc). A 6F catheter was advanced to the pulmonary artery bifurcation. A spiral configuration of 8 successful ablations was performed along the right and left pulmonary arteries (temperature >55°C; impedance reduction >10%; ablation duration >1 minute). The procedure was developed based on the anatomic location of nerves around the pulmonary arteries, the feasibility of catheter access to the pulmonary artery wall, and known parameters of radiofrequency energy delivery. For each ablation, the PDN catheter, on a 0.014″ guidewire, was positioned distal to the intended point of denervation. Withdrawal of the guidewire allowed the catheter to reform its resting conformation and apposed the electrode to the luminal aspect of the pulmonary artery. The bifurcation of the main pulmonary artery and the ostium of the basal segmental branches were used as proximal and distal anatomic markers for the procedure. Fluoroscopy and impedance were used to guide catheter positioning and confirm contact of the electrode with the pulmonary artery wall. Continuous monitoring of output, temperature, and impedance was used to monitor and optimize each ablation (Figure II in the Data Supplement). The sham procedure was performed using a dummy catheter that did not deliver radiofrequency energy. The first challenge of TxA_2_ was performed, and hemodynamic parameters were allowed to return to baseline. The catheter was advanced to the point of denervation and the generator activated; however, no radiofrequency energy was delivered. The second challenge of TxA_2_ was performed as previously described.

### Examination of the Pulmonary Artery After PDN

For histological examination of the pulmonary artery postablation, tissue was excised as previously described, and the pulmonary arteries dissected from surrounding tissue from the pulmonary valve distally. The luminal surface of the pulmonary artery was exposed via an anterior incision and tissue prepared en face. Ablation points were identified by the presence of blisters on the luminal aspect of the pulmonary artery. Sections were prepared as previously described and stained for hematoxylin and eosin, Alcian blue Elastin/van Gieson (ABEVG), S100 protein (Z0311, Dako), and α-smooth muscle actin (M0851, Dako).^14^

### Statistical Analysis

Data are expressed as mean±SEM. Differences between data sets were assessed by 2-way ANOVA with Sidak’s correction for multiple comparison or Student *t* test as appropriate in Prism 6.0 for Macintosh (GraphPad Software).

## Results

### Nerve Distribution of the Pulmonary Artery

**Figure 1.**
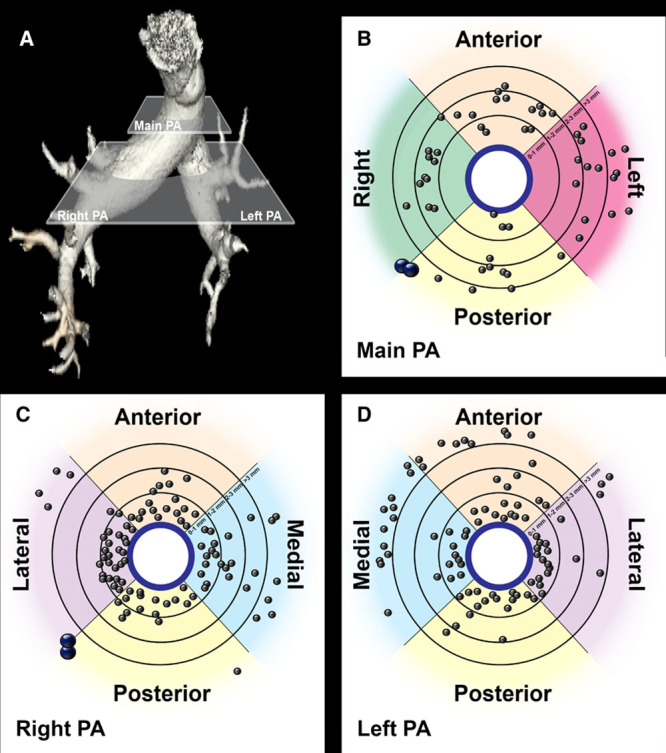
Pulmonary artery (PA) nerve distribution. **A**, Volume reconstruction of the pulmonary arteries. Main pulmonary artery plane and right and left pulmonary artery plane represent the levels at which histological sections were taken for evaluation of nerve distribution. **B**–**D**, Idealized representation of nerve distribution around the main (**B**) and right (**C**) and left pulmonary arteries (**D**). Gray points represent single nerves, and blue points represent nerve trunks (n=3).

To map nerve distribution, the pulmonary arteries (main pulmonary artery and left and right pulmonary artery) of 3 Yorkshire white swine were evaluated histologically. At the level of the main pulmonary artery (Figure 1A), there was circumferential distribution of nerves (Figure 1B) that were limited to the anterior and right side by the pericardial reflection and adventitia of the great vessels (Figure IC and IE in the Data Supplement). The majority of nerves were located between 1 and 3 mm from the endothelial aspect of the pulmonary artery wall and were of a greater diameter than those located more distally (Figure 1A; Figure IH and II in the Data Supplement). Distal to the pulmonary artery bifurcation nerves (Figure 1A) were distributed circumferentially around the right and left pulmonary artery. Nerves were greater in number, smaller in size, and located in closer proximity to the luminal aspect of pulmonary artery wall (<1 mm) than those at the level of the main pulmonary artery (Figure 1B–D; Figure IH and II in the Data Supplement). The number of nerves in close proximity to the right pulmonary artery was greater than the left pulmonary artery (Figure 1C and 1D). Discrete nerve bundles were identified >3 mm posterior and right of the main pulmonary artery and posterior and lateral of the right pulmonary artery (Figure 1B and 1C).

### TxA_2_ Induces Acute PH in a Porcine Model

**Figure 2.**
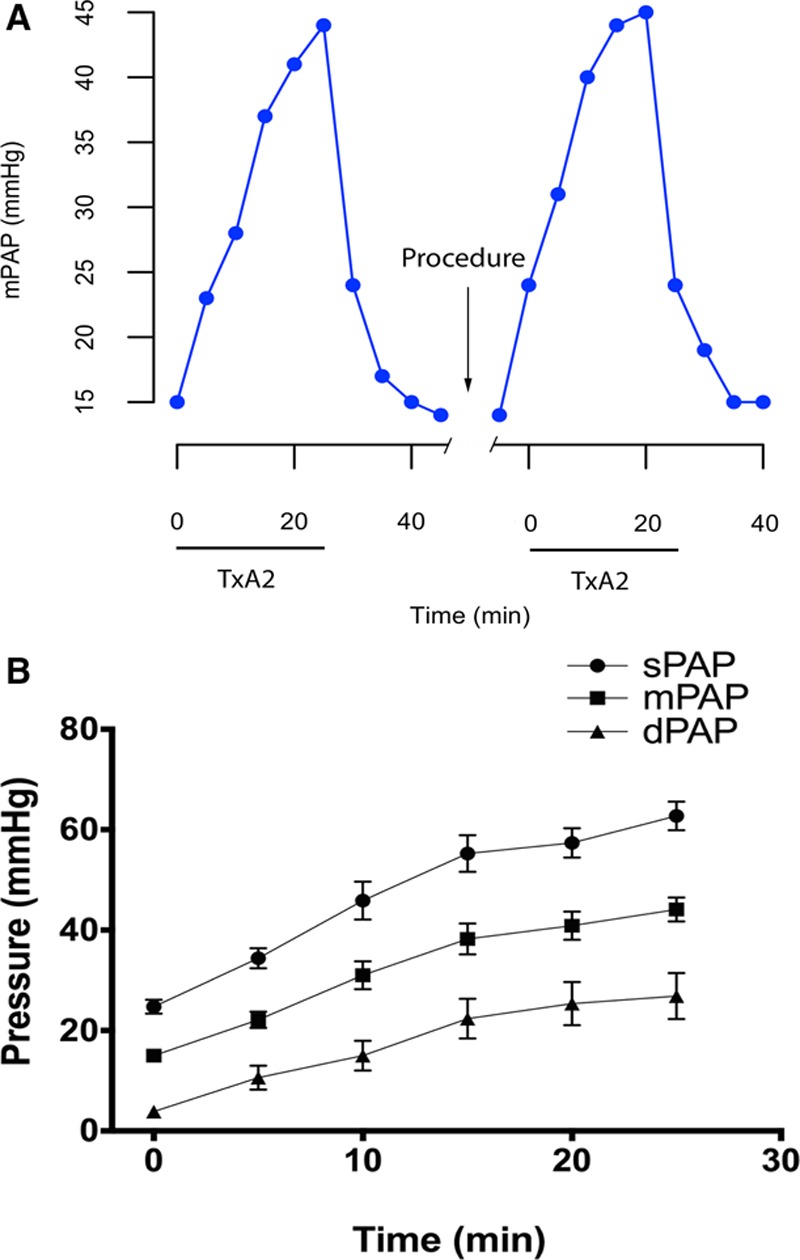
Experimental model of pulmonary hypertension. **A**, The dose of thromboxane A_2_ agonist (TxA_2_) was increased at 5-minute interval (0–37 μg/kg per hour) and then stopped. Once mean pulmonary artery pressure (mPAP) had returned to baseline, pulmonary artery denervation or a sham procedure was performed, and the TxA_2_ infusion was repeated. **B**, Stepped infusion of TxA_2_ gives a reproducible dose-dependent increase in systolic (sPAP), mean (mPAP) and diastolic PAP (dPAP) (n=8, mean±SEM).

**Figure 3.**
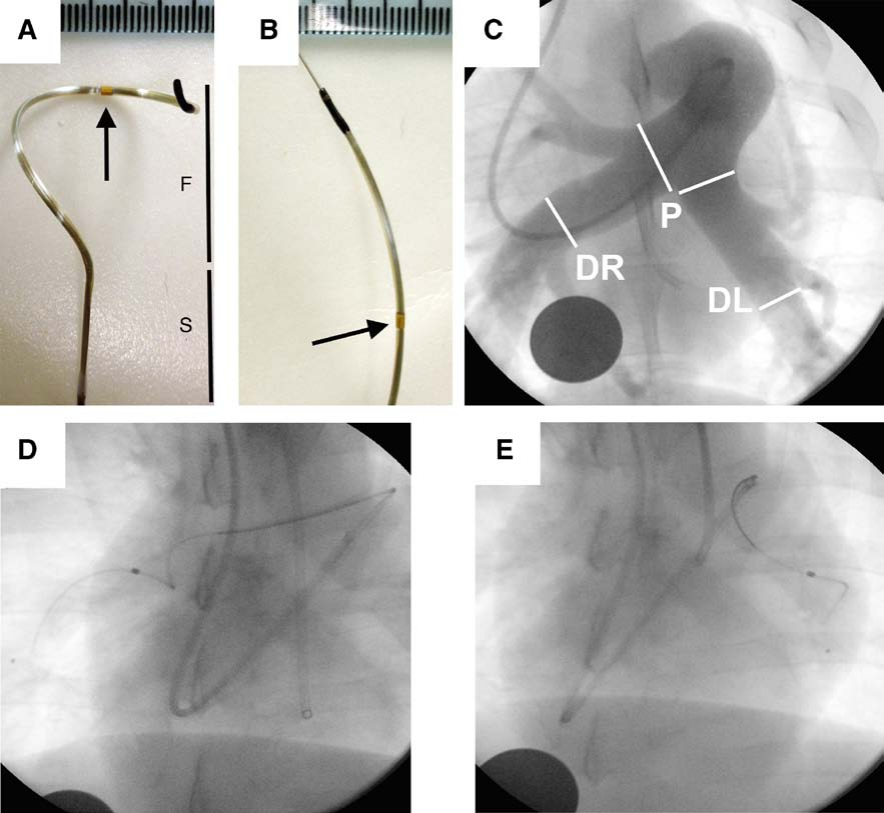
Pulmonary artery denervation (PDN) catheter and procedure. Prototype radiofrequency catheter (Medtronic Inc) off (**A**) and on (**B**) a 0.014″ guidewire (F indicates nitinol Spyral; S, catheter shaft; arrow, radiofrequency electrode; scale increment 1 mm). **C**, Pulmonary artery angiogram. White lines represent the proximal and distal boundaries for PDN procedure. Proximal (P) indicates pulmonary artery bifurcation; left distal (DL), ostium of the posterior artery; and right distal (DR), posterior descending artery (disc: 26 mm). Fluoroscopy of the PDN catheter in the right (**D**) and left pulmonary artery (**E**).

To determine the effect of PDN on PH, an acute TxA_2_ swine model of PH was established.^13^ Infusion of TxA_2_ led to a reproducible, dose-dependent rise in PAP (Figure 2A and 2B). Cessation of TxA_2_ infusion resulted in the PAP returning to baseline (Figure 2A). Repeated infusion of TxA_2_ increased PAP in a manner comparable with the initial infusion (Figure 2A), thereby establishing a robust model to test the efficacy of PDN (Figure 3).

### Acute Safety

Animals remained hemodynamically stable throughout the procedure. No adverse events were identified as a result of administration of TxA_2_ or PDN.

### PDN Reduces Mean PAP in an Acute Porcine Model of PH

**Figure 4.**
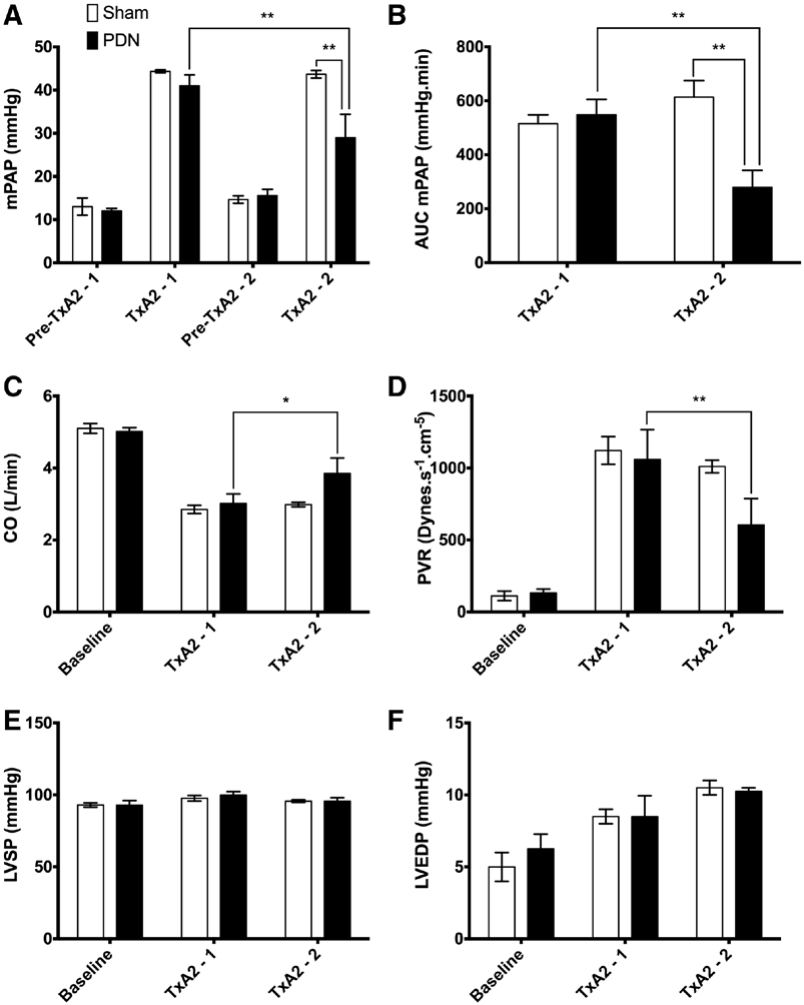
Experimental study hemodynamics. **A**, Mean pulmonary artery pressure (mPAP) at baseline and maximum thromboxane A_2_ agonist (TxA_2_) pre (TxA_2_-1) and post (TxA_2_-2) procedure for sham- and pulmonary artery denervation (PDN)-treated animals. **B**, Area under the curve (AUC) of time–pressure for PDN- and sham-treated animals during the pre and post procedure TxA_2_ challenges, cardiac output (CO; **C**), pulmonary vascular resistance (PVR; **D**), LVSP (**E**), and LVEDP (**F**) at baseline and maximum TxA_2_ pre and post procedure for sham- and PDN-treated animals. PDN, n=5; Sham, n=3; mean±SEM. LVEDP indicates left ventricular end diastolic pressure; and LVSP, left ventricular systolic pressure. *P<0.05, **P<0.01, 2-way ANOVA with Sidak’s correction for multiple comparisons.

There was no significant difference in hemodynamic response to the first infusion TxA_2_ between groups selected for PDN or Sham treatment (Figure 4A–4F). Both PDN and Sham-treated animals developed PH in response to TxA_2_ as demonstrated by mean PAP (mPAP) of >40 mm Hg (Figure 4A). Consistent with the observed change in mPAP, CO was decreased and PVR increased during the induction of PH in both groups (Figure 4C and 4D). Maximum mPAP was comparable between first and second TxA_2_ challenge in the Sham group; however, PDN resulted in a reduction in mPAP from first to second TxA_2_ challenge in the PDN group and between the Sham and PDN group during the second TxA_2_ challenge (Figure 4A). The difference in mPAP was further emphasized by the reduction of area under the curve of mPAP against time from first to second TxA_2_ challenge in the PDN group and between the Sham and PDN group during the second TxA_2_ challenge (Figure 4B). Consistent with the pressure changes identified, PDN resulted in improved CO and PVR (Figure 4C and 4D). PDN did not alter left ventricular systolic or left ventricular end diastolic pressure (Figure 4E and 4F).

### PDN Induces Acute Histological Changes and Reduces Adventitial Nerve Staining

**Figure 5.**
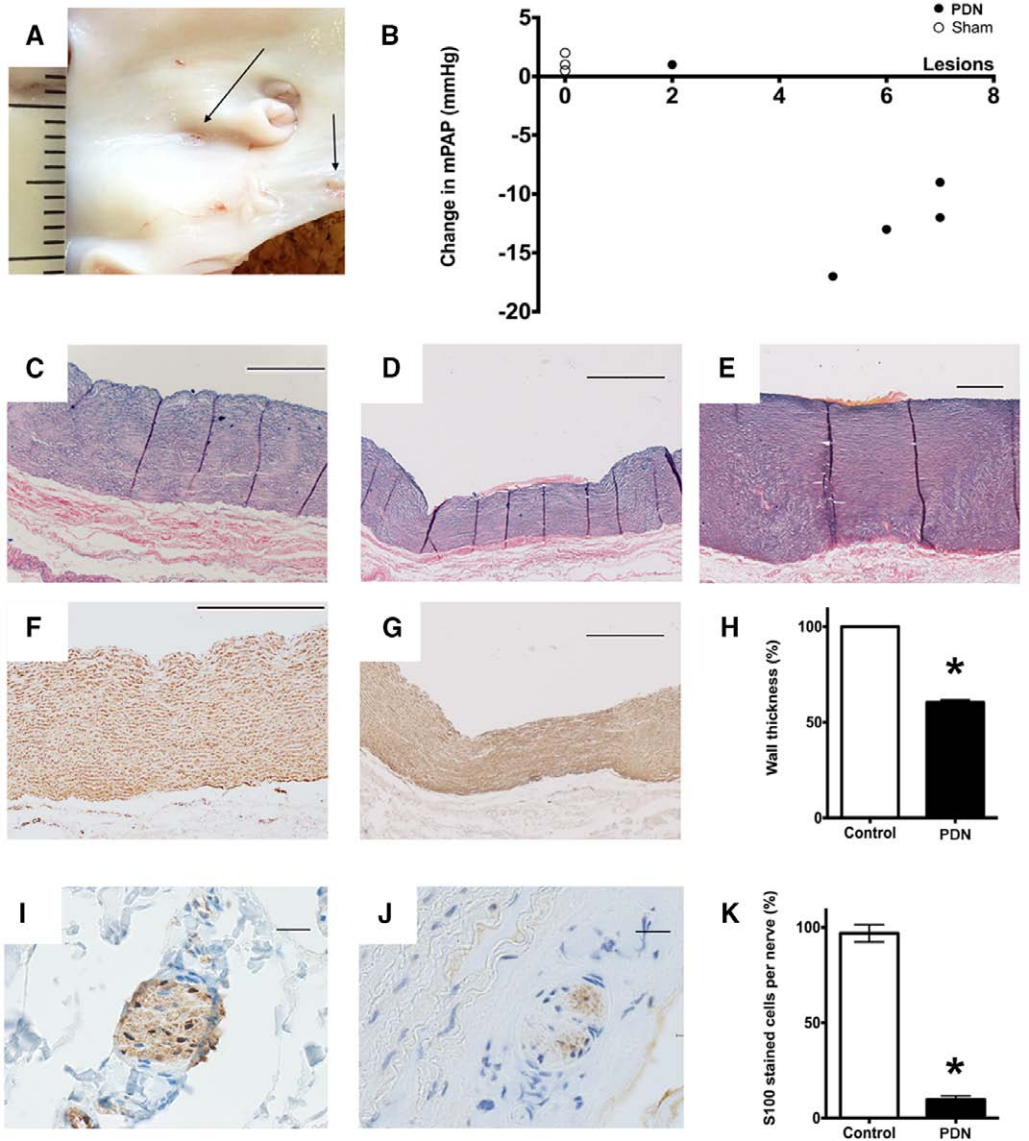
**A**, En face preparation of the pulmonary artery. Arrows indicate denervation lesions in the right pulmonary artery. **B**, Change in mean pulmonary artery pressure (mPAP) between first and second infusion of thromboxane A_2_ agonist (TxA_2_) plotted against the number of visible ablation lesions. **C**–**E**, Alcian blue Elastin/van Gieson (ABEVG)–stained artery sections (scale bar: 500 μm). **C**, Control pulmonary artery, (**D**) denervation lesion in the pulmonary artery, and (**E**) denervation lesion in the aorta. **F** and **G**, Smooth muscle actin–stained pulmonary artery sections (scale bar: 500 μm). **F**, Control and (**G**) denervation lesion. **H**, The acute effect of pulmonary artery denervation (PDN) on wall medial thickness. **I** and **J**, S100 protein–stained sections showing nerves in the pulmonary artery adventitia (scale bar 20 μm). **I**, Control and (**J**) denervation lesion. **K**, The acute effect of PDN on nerve S100 protein staining (n=5, mean±SEM, *P<0.05, paired Student *t* test).

Ablation lesions were visible on the luminal aspect of the pulmonary artery of each animal in the PDN group (Figure 5A), and the number of lesions correlated with hemodynamic response (Figure 5B). Microscopic examination of denervation lesions identified intimal disruption and thrombus (Figure 5C and 5E) with linear elastic laminae (fixed within area of thermal damage), reduced medial thickness (Figure 5C, 5D, 5F, and 5G), and altered adventitial architecture (Figure 5C and 5E). There was also a reduction in the expression of the nerve-associated S100 protein (Figure 5I and 5J). The observed acute histological changes were consistent between animals and between lesions (Figure 5H and 5K).

## Discussion

This study maps the location and depth of the nerve distribution of the pulmonary arteries and demonstrates, using a prototype radiofrequency catheter and generator, that PDN alters the structure of nerves supplying the pulmonary arteries and results in a dose-dependent improvement in pulmonary hemodynamics. The acute reduction in PAP achieved with PDN suggests that there is a sympathetic contribution to vasoconstriction in PH that may be therapeutically tractable.

The distribution and function of nerves supplying the pulmonary arteries has been documented in a range of animals^15^; however, data relating anatomic location to nerve depth has not previously been reported. We identified nerve fibers and trunks at the level of the main pulmonary artery and left and right pulmonary arteries with marked variation in vessel wall thickness, nerve depth, and nerve diameter at each level. The depth of histological changes induced by radiofrequency energy delivery varied with anatomic location and wall thickness. In arteries with a greater medial component, such as the aorta or proximal pulmonary artery, there was little or no histological effect of radiofrequency energy delivery on nerve S100 protein staining, indicating that the location and technical parameters of energy delivery are critical to successful PDN. Further anatomic and physiological investigation of nerve distribution and function is clearly required to improve understanding of the therapeutic mechanism of action and enable procedural optimization.

Inflammation, cellular proliferation, and vasoconstriction all drive disease pathology in PAH^16^; however, the contribution of these underlying factors to the burden of disease in an individual patient is not known. Elevated plasma norepinephrine,^3,4^ increased muscle sympathetic nerve activity,^5,6^ and increased vessel sympathetic nerve endings^7^ in patients with idiopathic PAH have all been described, thus implicating sympathetic activity in disease. Modulation of the neurohormonal axis in PAH, using pharmacological^17^ and device-based therapies^11^ has shown benefit in experimental and early human studies.^12^ Further work is required to understand the role in disease pathology and establish the therapeutic mechanism. Previous PDN studies have demonstrated efficacy using a balloon occlusion model,^8,11^ however, the mechanism by which PH is induced in that model remains controversial. Balloon occlusion is thought to cause an increase in PAP through activation of a pulmo-pulmonary baroreflex,^9^ but the extent to which this reflex contributes to human disease is unknown. Vasoconstriction is a fundamental mediator of disease pathology in PAH. We therefore used a vasoconstriction-based model of PH^13^ and have demonstrated that PDN reduced PAP and PVR and increased CO, with a limited reduction of systemic blood pressure. Our findings suggest that the innervation of the pulmonary vasculature contributes to PH in disease models beyond balloon occlusion. Although our findings do not fully replicate the complete prevention^11^ or reversal^12^ of PH previously reported, the improvement in pulmonary hemodynamics achieved would be clinically meaningful. Although PDN has shown promising early results, it is critically important that the mechanistic effects of intervention are fully evaluated in the context of chronic disease.

### Limitations

PH in the TxA_2_ model is induced by acute vascoconstriction and not by progressive pulmonary vascular remodeling. The acute nature of the experiment precluded the evaluation of long-term effect on pulmonary hemodynamics and vessel histology. Although vasoconstriction plays a role in disease pathology and is a therapeutic target for the treatment of PAH, the improved hemodynamics demonstrated in this study may not translate directly to human disease.

### Conclusions

In an acute vasoconstriction-based model of PH, we have provided a detailed description of the distribution of nerves in the proximal pulmonary vasculature. We have also demonstrated the efficacy of PDN and described the associated histological and biochemical changes. Further studies are required to optimize the PDN procedure and to investigate the safety and the long-term efficacy of intervention before large clinical trials in patients are considered.

## Acknowledgments

We would like to acknowledge Ms Tracy Sanderson (Department of Histopathology, Sheffield Teaching Hospitals NHS Trust) for assistance with preparation of histological sections, and Dr Rachael Elder (Department of Chemical and Biological Engineering, University of Sheffield), Mr James Iremonger, and Mr Adam Braithwaite (Department of Cardiovascular Science, University of Sheffield) for assistance with representation of nerve distribution data.

## Sources of Funding

Medical Research Council Confidence in Concepts (Drs Rothman, Gunn, and Lawrie: MC/PC/12022) and Clinical Research Training Fellowship (Dr Rothman: MR/K002406/1) awards, and a British Heart Foundation Senior Basic Scientist Fellowship (Dr Lawrie: FS/13/48/30453) award. Catheters and generator were provided by Medtronic Inc via an External Research Program award (Dr Rothman).

## Disclosures

Dr Rothman has received research support from Medtronic Inc and consulting fees from SoniVie Ltd. W. Chang is an employee of Medtronic Inc. The other authors report no conflicts.

## Supplementary Material
