# Supplementary material for: Pulmonary Artery Denervation Reduces Pulmonary Artery Pressure and Induces Histological Changes in an Acute Porcine Model of Pulmonary Hypertension
Source: Circ Cardiovasc Interv. 2015 Nov 17;8(11):e002569. doi: 10.1161/CIRCINTERVENTIONS.115.002569 (PMC4648184; doi:10.1161/CIRCINTERVENTIONS.115.002569)
Supplement: Supplementary file 1 [file hcv-8-e002569-s001.docx]

What is known:

• Sympathetic tone is increased in patients with pulmonary arterial hypertension.

• Increased sympathetic tone has been associated with elevated pulmonary artery pressures.

What the study adds:

• Documents the distribution and depth of nerves surrounding the pulmonary

artery in an animal model.

• Demonstrates the acute effect of radiofrequency energy delivery to the

pulmonary artery wall.

• Demonstrates the efficacy of pulmonary artery denervation in an acute model

of pulmonary hypertension.
