# Supplementary figures and images for: Pulmonary Artery Denervation Reduces Pulmonary Artery Pressure and Induces Histological Changes in an Acute Porcine Model of Pulmonary Hypertension
Source: Circ Cardiovasc Interv. 2015 Nov 17;8(11):e002569. doi: 10.1161/CIRCINTERVENTIONS.115.002569 (PMC4648184; doi:10.1161/CIRCINTERVENTIONS.115.002569)

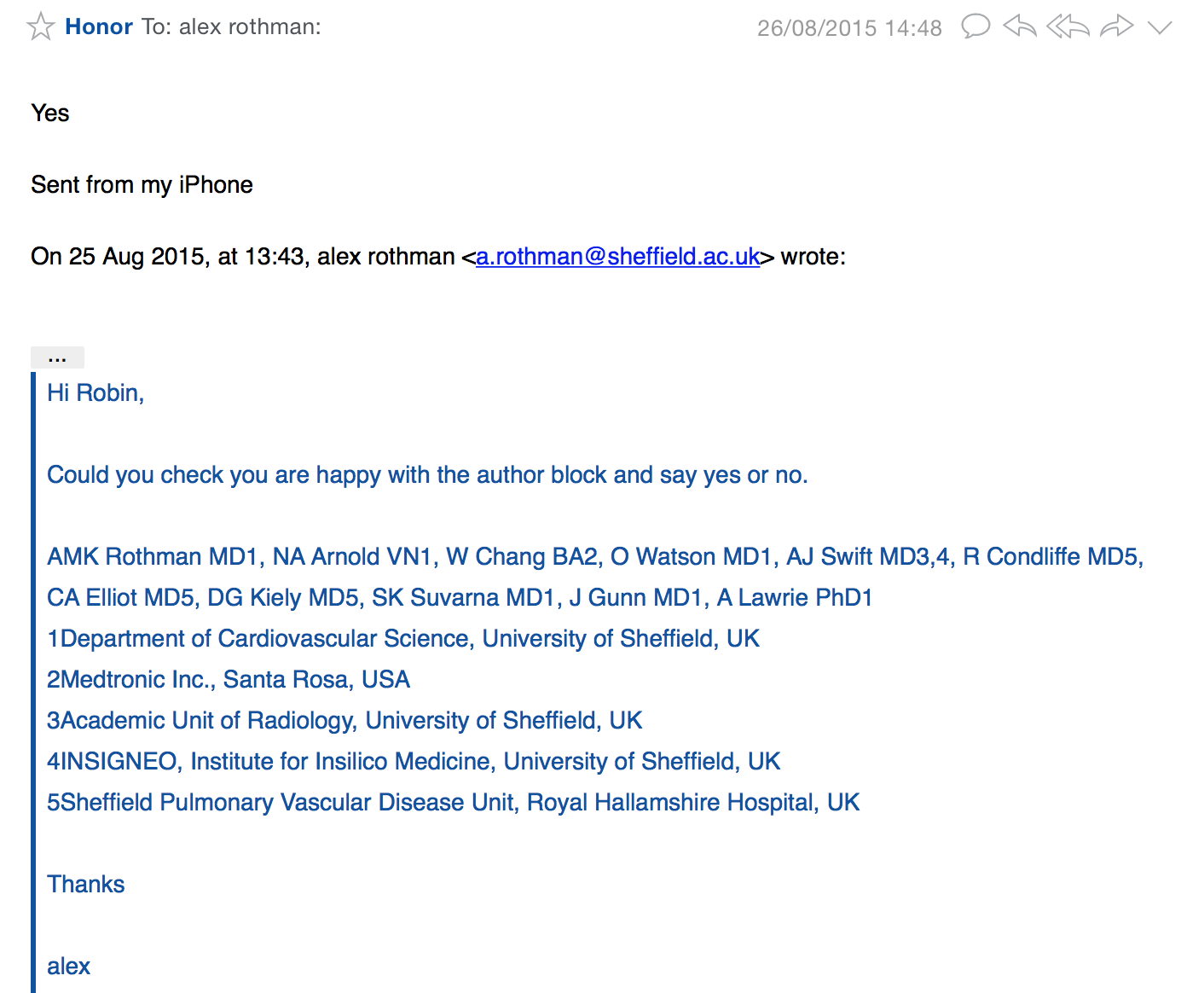

Supplement: Supplementary file 2 [file hcv-8-e002569-s002.png]

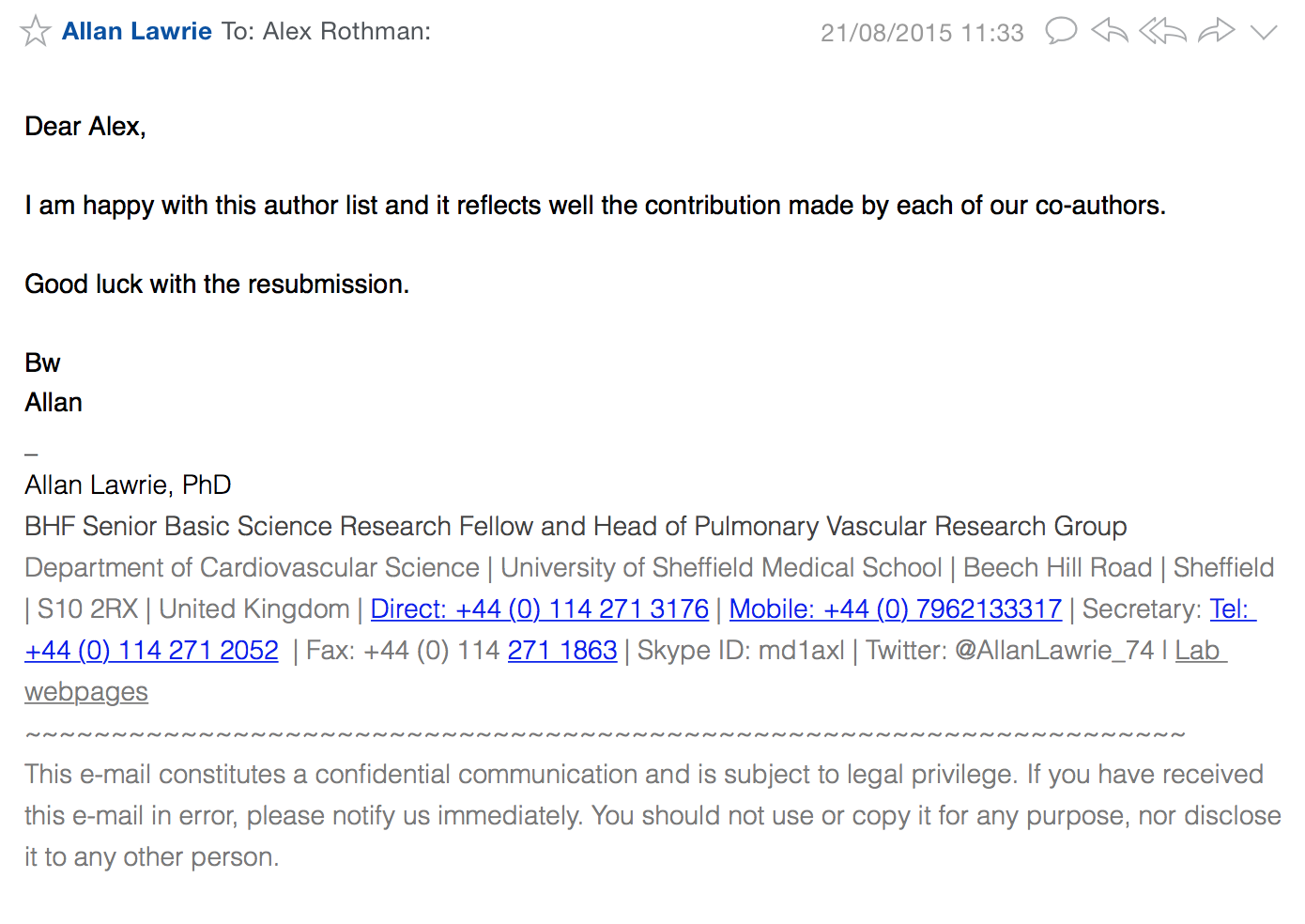

Supplement: Supplementary file 3 [file hcv-8-e002569-s003.png]

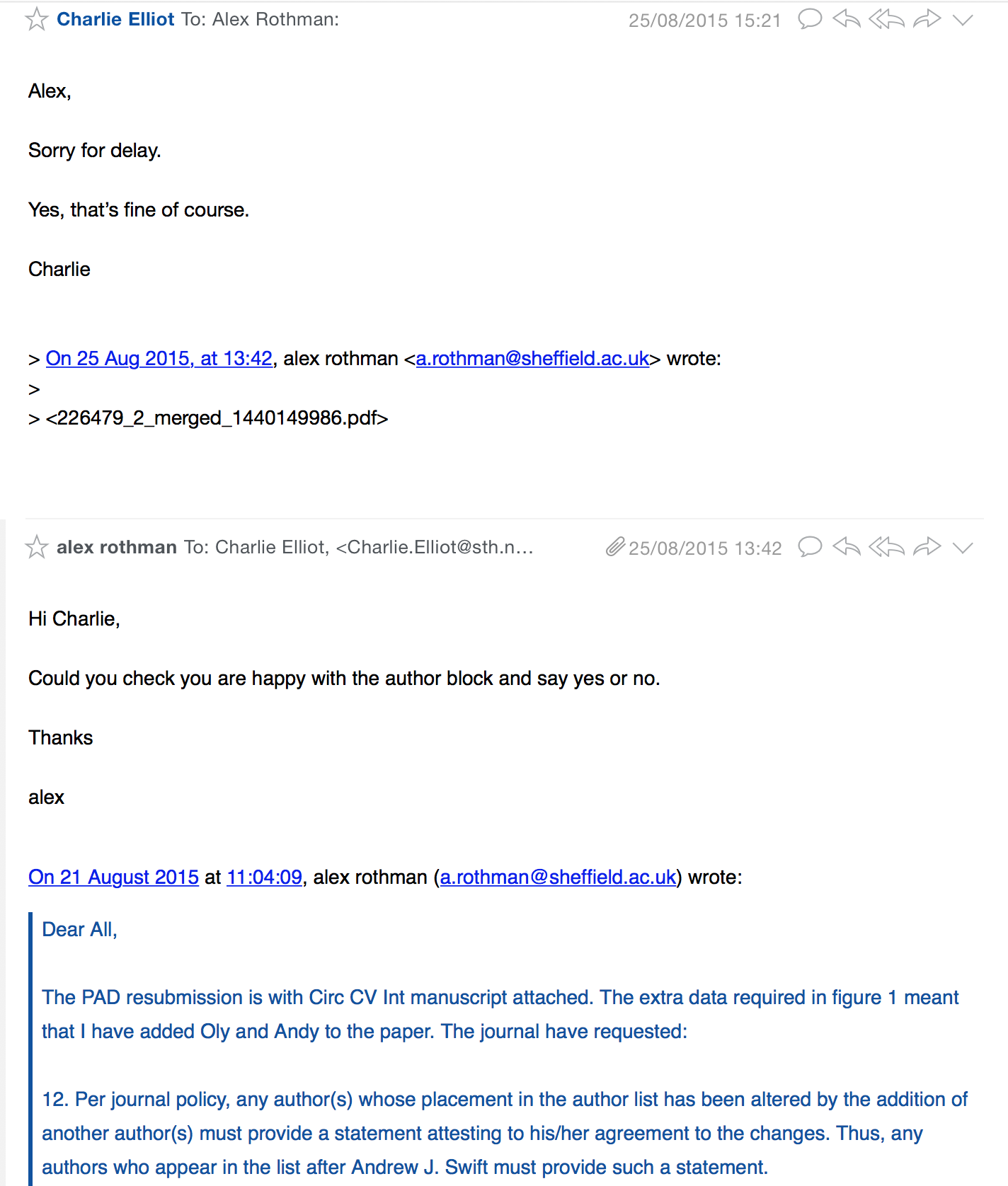

Supplement: Supplementary file 4 [file hcv-8-e002569-s004.png]

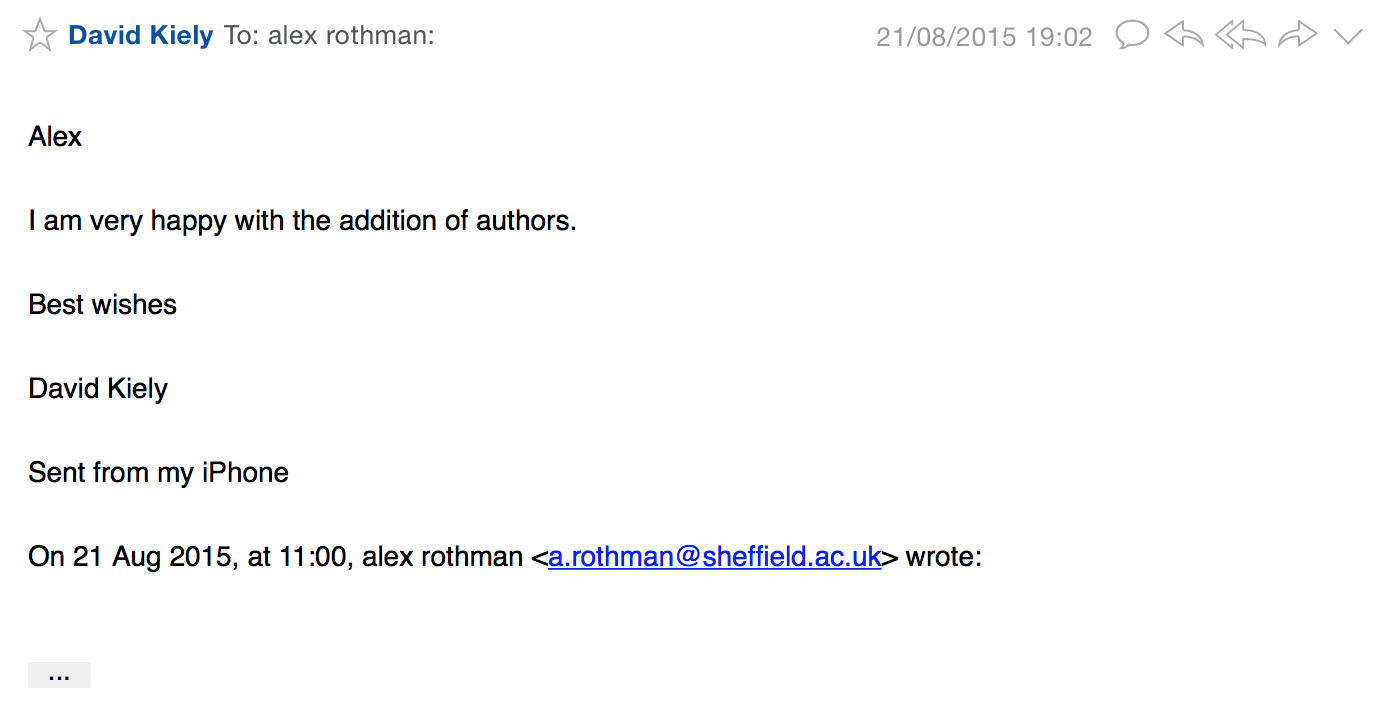

Supplement: Supplementary file 5 [file hcv-8-e002569-s005.png]

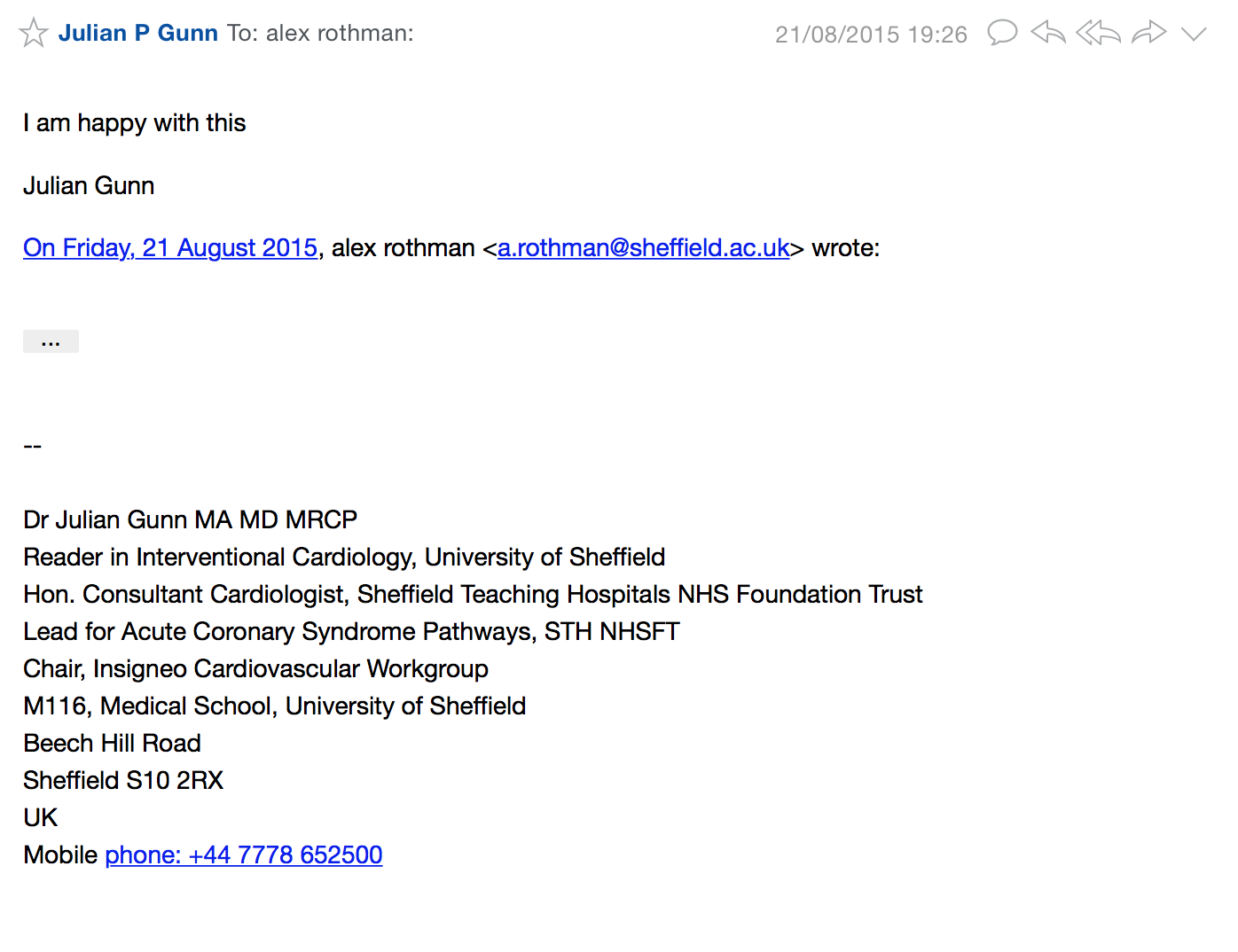

Supplement: Supplementary file 6 [file hcv-8-e002569-s006.png]

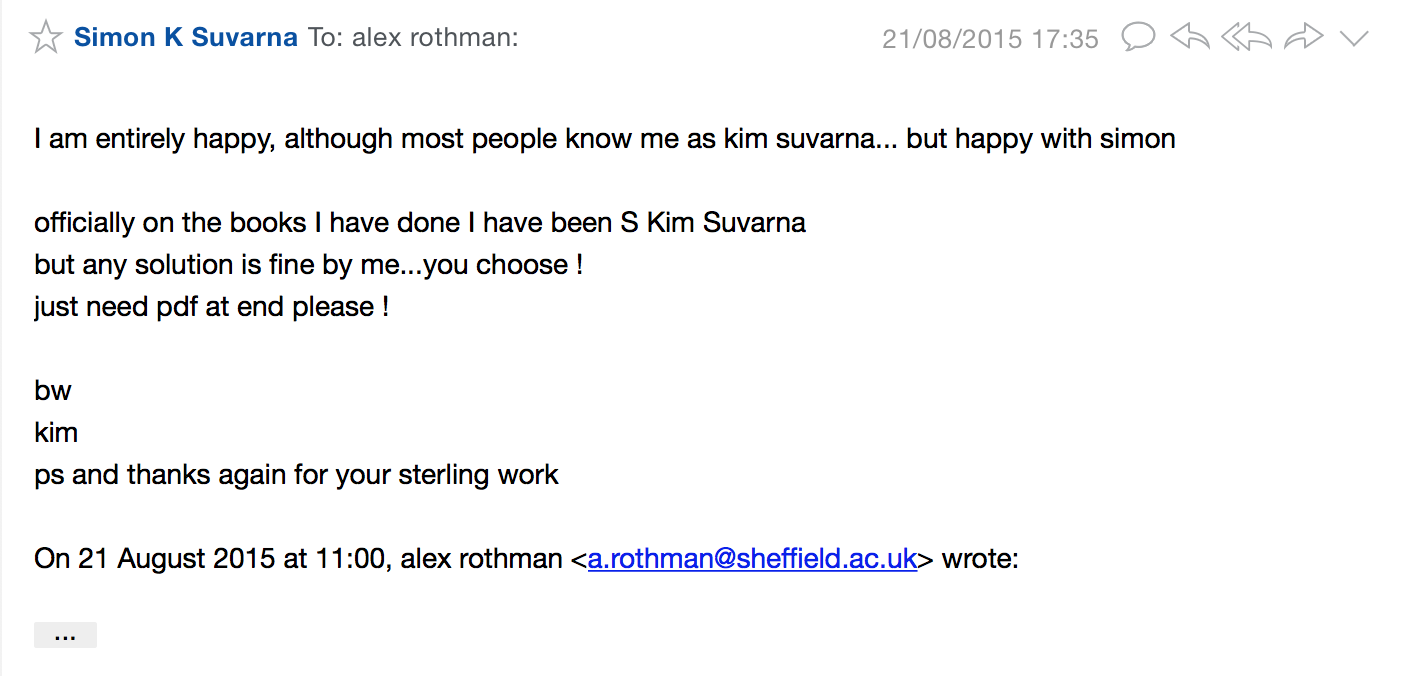

Supplement: Supplementary file 7 [file hcv-8-e002569-s007.png]
