# Supplementary material for: Pulmonary Artery Denervation Reduces Pulmonary Artery Pressure and Induces Histological Changes in an Acute Porcine Model of Pulmonary Hypertension
Source: Circ Cardiovasc Interv. 2015 Nov 17;8(11):e002569. doi: 10.1161/CIRCINTERVENTIONS.115.002569 (PMC4648184; doi:10.1161/CIRCINTERVENTIONS.115.002569)
Supplement: Supplementary file 8 [file hcv-8-e002569-s008.pdf]

SUPPLEMENTAL MATERIAL

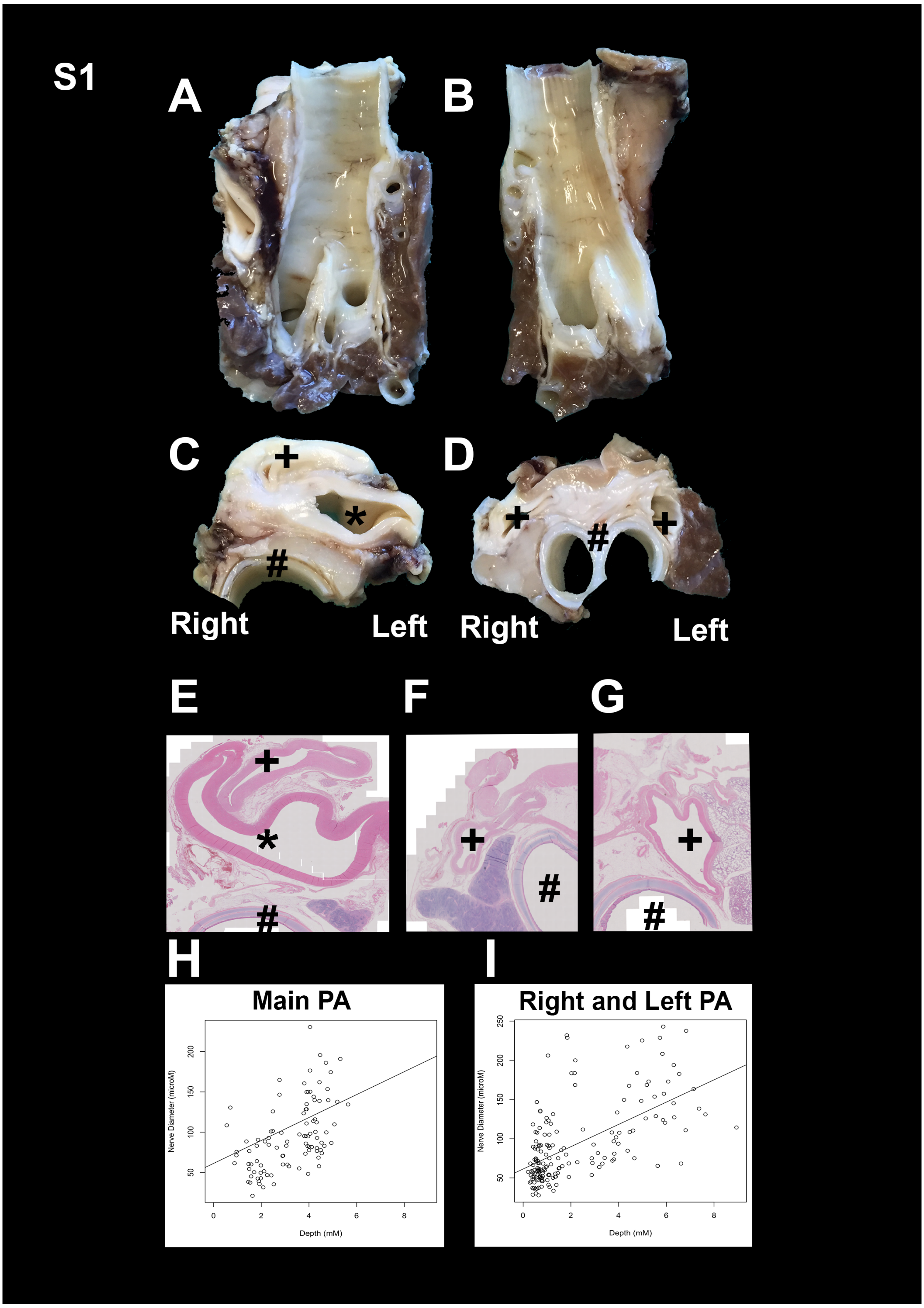

Supplemental Figure 1: Pulmonary artery anatomy, histology and nerve distribution: A and B: Excised blocks divided along the trachea to allow preparation of axial sections. C-D: Axial sections at the level of the main pulmonary artery (C) and right

and left pulmonary arteries (D, + pulmonary artery, * aorta and # trachea/carina). E-F: Representative axial sections at the level of the main pulmonary artery (E) and right (F) and left (G) pulmonary arteries (+ pulmonary artery, * aorta and # trachea/carina). H-I: Nerve diameter (μm) and depth from luminal surface of the pulmonary artery (mm) at the level of the main pulmonary artery (H) and right and left pulmonary arteries (I).

S2

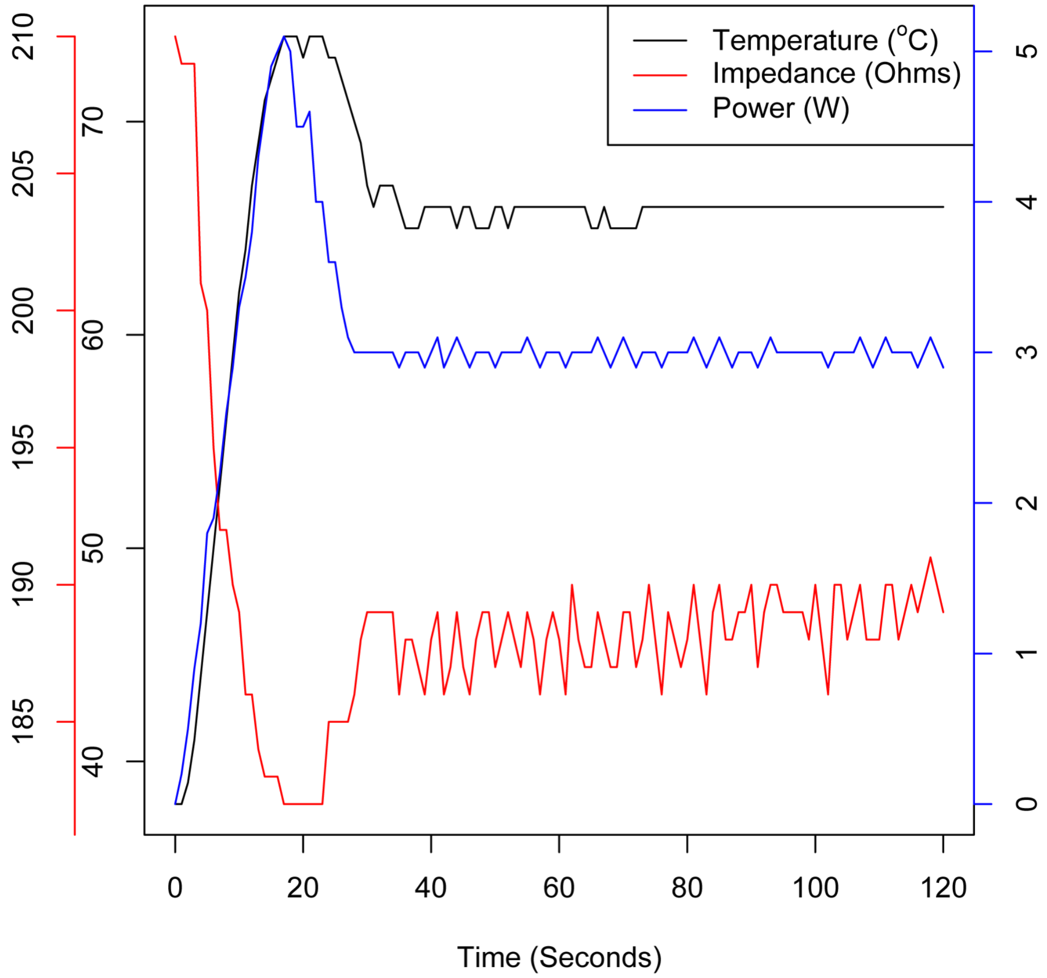

Supplemental Figure 2: Representative temperature (°C, black), impedance (Ohms, red) and power (W, blue) data from a single pulmonary artery ablation.
